# Primary Prophylaxis of Overt Hepatic Encephalopathy: Is It Time to Consider It?

**DOI:** 10.3390/jcm12123903

**Published:** 2023-06-07

**Authors:** Jessica Faccioli, Silvia Nardelli, Stefania Gioia, Oliviero Riggio, Lorenzo Ridola

**Affiliations:** Department of Translational and Precision Medicine, Sapienza University of Rome, 00185 Rome, Italy; jessica.faccioli@uniroma1.it (J.F.); silvia.nardelli@uniroma1.it (S.N.); stensgioia@hotmail.com (S.G.); oliviero.riggio@uniroma1.it (O.R.)

Hepatic encephalopathy (HE) represents one of the most frequent complications of liver cirrhosis and one of the most debilitating clinical manifestations of liver disease due to the accumulation of toxic substances in the blood and central nervous system. Most of these substances are produced by bacterial fermentation of dietary protein, and among these, ammonium plays a major role [1].

The pathogenesis of HE is multifactorial and multiorgan. Ammonia exerts its deleterious effects through multiple pathways, including cellular swelling, inflammation, oxidative stress, mitochondrial dysfunction, and neurohinibition. In addition, there is a relationship between ammonium level and severity of HE: higher ammonium values are associated with more severe episodes.

Neuroinflammation and oxidative stress, resulting from increased bacterial translocation, are also frequently found in cirrhotic patients and play a pathogenic role through blood-brain barrier impairment. The increased bacterial translocation is caused by a condition called dysbiosis, characterized by the presence of decreased bacterial diversity and increased pathogenic species such as Proteobacteria and Streptococcaceae. Finally, in recent years, the relationship between muscular alterations and HE has been studied. In fact, skeletal muscle may play a compensatory role in ammonia clearance because of liver damage. So, muscle depletion may favor ammonia accumulation and HE development [2].

HE has a wide spectrum of neurological and/or psychiatric symptoms, and they are classified in two forms based on their severity according to West Haven criteria: “covert” and “overt”.

“Overt” HE (OHE) includes patients with temporo-spatial disorientation, inappropriate behaviors, agitation, or coma. “Covert” HE includes “minimal” HE (MHE) and grade I HE. Patients with grade I HE have subtle cognitive and/or behavioral changes, while those with MHE are completely asymptomatic but have neuropsychological changes detectable with psychometric tests (1). These patients have a worse quality of life, a higher risk of progression to OHE, an increased risk of falls and traffic accidents, and sleep and somatosensory alterations [3].

Treatments used for HE act on intestinal production, absorption, and elimination of ammonium. Among these drugs, non-absorbable disaccharides are considered the standard-of-care for HE treatment. In fact, lactulose has ionizing, prebiotic, and laxative effects, and through these mechanisms, it can reduce plasma ammonium. Rifaximin, a non-absorbable antibiotic, has a direct action on ammonium-producing enteric bacteria, is safe and well tolerated, and represents the second most widely used drug in HE treatment [4].

Primary prophylaxis of HE involves the use of strategies to prevent the development of a first episode of OHE in high-risk patients.

The development of HE in patients with acute variceal bleeding (AVB) has an incidence between 16.9% and 40%.

Bleeding is one of the main precipitants of HE and is due to the absorption of toxic substances from blood proteins. This condition is associated with increased morbidity and mortality. The efficacy of prophylactic treatment in this group of patients is based on strong evidence in the literature.

The randomized controlled trial vs. placebo by Sharma et al. confirmed that the incidence of HE was lower in patients with AVB treated with oral lactulose for five days at a dosage of 30–60 mL divided into two-to-three doses to achieve two or three daily evacuations of soft stools (14% vs. 40%, *p* = 0.02) [5].

These results confirmed previous data in which cirrhotic patients with upper gastrointestinal bleeding (UGB) of any etiology were treated with lactulose; both lactulose therapy and disease severity were significantly associated with the occurrence of HE, and its administration was safe and well tolerated [6].

On the contrary, another randomized controlled clinical trial showed that lactulose therapy did not reduce the incidence of HE after AVB and that high a Child–Pugh class and diarrhea were predictive for the development of HE. So, the authors concluded that in these patients, unnecessary laxatives should be avoided given their prophylactic ineffectiveness [7].

Recent EASL guidelines on HE management indicate that rapid removal of blood from the gastrointestinal tract can be used to prevent the development of HE [1].

HE is a frequent complication after transjugular intrahepatic porto-systemic shunt (TIPS) placement, with an incidence between 35% and 50%.

The study by Schepis et al. showed that the use of stents with a smaller diameter reduced the incidence of HE during the first year after TIPS placement (27% vs. 54%) with equal efficacy [8]. However, the benefit of using these stents was not confirmed in all studies. In the study by Riggio et al., their use resulted in a higher incidence of portal hypertension complications without significant benefit to HE incidence [9].

Given the high incidence of post-TIPS HE, there are several trials on pharmacological prophylaxis of this condition using non-absorbable disaccharides and Rifaximin. However, to date, evidence of effective measures is weak.

A randomized controlled trial comparing lactitol and Rifaximin vs. placebo showed no differences in HE incidence one month after the procedure [10].

A more recent trial showed that Rifaximin at a dosage of 600 mg twice daily from 14 days before to 6 months after TIPS reduced the incidence of HE (34% vs. 53%). However, if only patients without a history of HE were considered, this difference would not reach statistical significance. Moreover, these results are limited to a certain etiology of cirrhosis (alcohol) and over a short period of time (six months) [11].

So, the most effective strategy to prevent this complication is the careful selection of patients to identify possible risk factors. In accordance with these considerations, patients in whom TIPS placement should be considered with caution are those with advanced age and liver cirrhosis (Child-Pugh score > 12), those with a history of previous overt HE or with MHE, or patients with reduced muscle mass. The risk of HE may outweigh the potential benefit of the procedure in patients possessing these risk factors [12].

The radiological technique can also influence the risk of post-TIPS HE. For example, it would be desirable to avoid achieving a too low post-TIPS porto-systemic gradient (PPG < 5 mmHg) and the use of large stents (>10 mm), while concurrent large spontaneous portosystemic shunt embolization is effective to prevent post-TIPS HE without increasing complications [13].

MHE is a known risk factor for progression to OHE and mortality. In the prospective study by Hartmann et al., during a mean follow-up of 29 months, patients with MHE had a 3.7-fold increased risk of developing HE (*p* = 0.002) and more episodes of HE during follow-up (56% vs. 8%, *p* < 0.001), compared with patients without cognitive decline [14]. Among patients with TIPS, the study by Nardelli et al. showed that there was a significant difference in the incidence of post-TIPS HE between patients with and without MHE (*p* = 0.0003), which was greater in patients with pre-existing cognitive decline [15].

Given these premises, numerous studies have been conducted on MHE treatment, but in many of these, the endpoint was an improvement in psychometric testing.

The randomized trial by Watanabe et al. vs. placebo showed that 45 mL/day of lactulose for 8 weeks was able to eliminate MHE in 50% of treated subjects, compared with 15% of untreated patients [16].

Other studies have confirmed the effectiveness of therapy with non-absorbable disaccharides, Rifaximin, and prebiotics on clinical complications of MHE, such as the risk of falls [17], driving performance [18], and sleep disturbances [19].

Patients with MHE often have dysbiosis, which is characterized by an increase in Enterobacteriaceae and *Staphylococcus* spp. species [20]. Administration of probiotics/symbionts in these patients has shown a positive effect on performance on psychometric tests without differences in OHE incidence [21].

Although there is no robust evidence regarding the reduction of HE incidence after MHE therapy, it may be thought that its resolution may have a positive effect. For this reason, EASL guidelines recommend screening for MHE in all cirrhotic patients and its treatment with non-absorbable disaccharides [1].

Among the new precipitants of HE, muscle alterations play an important role. In malnourished cirrhotic patients, it is common to find sarcopenia, which is a reduction in muscle mass, and the presence of fat between and within muscle fibers, a condition known as mysteatosis. These conditions are more frequent in patients with severe disease, advanced age, a lower BMI, and a lower protein intake [22], and they are associated with a worse prognosis and several complications of liver cirrhosis, including HE [23].

Sarcopenia and myosteatosis can improve after TIPS placement, regardless of liver function and in parallel with cognitive gain (both minimal and overt); this supports a causal relationship between muscle changes and HE [24].

The study published by Gioia et al. showed that muscle tissue improved after TIPS and it was associated with a lower incidence of HE [25].

In light of these data, it may be assumed that improvement of nutritional status, and thus muscle changes, can be a primary prophylaxis strategy for HE. To date, data on this topic are poor.

The randomized trial by Maharshi et al. showed that nutritional intervention (30–35 kcal/kg/day and 1.0–1.5 g vegetable protein/kg/day) for 6 months improved MHE compared with the usual diet (71.1% vs. 22.8%; *p* = 0.001) and reduced OHE incidence (10% vs. 21.7%; *p* = 0.04) [26]. This purpose can be achieved through physical activity. Exercise in cirrhotic patients provides a wide range of benefits, including increased muscle mass and muscle function [27,28]. However, there are no studies on the prevention of HE after physical exercise.

Recently, there has been growing interest in the therapeutic role of albumin. In the ANSWER trial, long-term albumin administration improved the management of grade 2 or 3 ascites and reduced the incidence of complications of liver cirrhosis, including HE [29], while a meta-analysis showed that it could have a beneficial effect on survival after HE in addition to standard therapy [30]. Our group also recently demonstrated that administration of albumin for one month after TIPS placement resulted in a lower incidence of HE compared with the literature data, opening a future perspective for the use of this molecule [31].

However, further clinical studies are needed to confirm the positive effects of long-term administration of albumin, the target population, the optimal dosage and frequency of administration, and discontinuation.

The evidence described draws attention to the importance of identifying conditions that are risk factors for the development of HE and the positive effect that preventive therapies can have in reducing this risk.

## Data Availability

Data sharing is not applicable to this article as no new data were created or analyzed in this study.
